# Management of the primary malignant mediastinal germ cell tumors: experience with 54 patients

**DOI:** 10.1186/1746-1596-9-33

**Published:** 2014-02-19

**Authors:** Ying Liu, Zhou Wang, Zhong-Min Peng, Yang Yu

**Affiliations:** 1 Department of Thoracic Surgery, Shandong Provincial Hospital affiliated to Shandong University, 324 Jingwu Road, Jinan, 250021 Shandong Province, China

**Keywords:** Mediastinum, Nonseminomatous germ cell tumor, Seminoma

## Abstract

**Background:**

Primary malignant mediastinal germ cell tumor (PMMGCT) is rare and sometimes the prognosis of the patients with PMMGCT is not very satisfactory.

**Methods:**

A total of 54 patients with PMMGCT in a follow-up from 1990 to 2009. We evaluated the role of the surgical treatment and the effect of multimodality treatment strategy for patients with PMMGCT.

**Results:**

Fifty-two patients underwent surgical resections, while the other two patients just received chemoradiotherapy. Among the 52 patients, 28 cases received preoperative adjuvant therapy and 24 cases underwent surgery as initial treatment; 30 cases with complete resections, 18 cases with partial resections and 4 cases with only biopsies. There was no perioperative mortality. Histopathologic results revealed 18 cases of seminomas and 36 cases of nonseminomatous germ cell tumors (NSGCT). The last follow-up showed that 17 patients were alive, including 11 patients with seminoma and 6 patients with NSGCT. The 5-year overall survival rate of patients with seminomas was 87.7%. The 3-year and 5-year overall survival rates of patients with NSGCT were 47.4% and 23.0%, respectively.

**Conclusions:**

It could be concluded that a complete surgical resection of PMMGCT after chemoradiotherapy showed favorable long-term survival. Patients with pure seminomas have a better prognosis compared with that with NSGCT.

**Virtual slides:**

The virtual slides for this article can be found here: http://www.diagnosticpathology.diagnomx.eu/vs/1676987232116837.

## Introduction

Primary extragonadal germ cell tumors (GCT) are rare and account for only 1% to 5% of all germ cell malignancies [1,2]. The most common extragonadal sites are the mediastinum and retroperitoneum [3]. These extragonadal germ cell tumors histologically contain the same components as their gonadal counterparts, but may have different biologic behaviors, clinical characteristics and inferior overall prognoses [4]. Furthermore, primary malignant mediastinal germ cell tumors (PMMGCT) are rather rare and represent only 1% to 4% of all mediastinal tumors [5]. PMMGCT can be divided into two broad groups: seminomas and nonseminomatous germ cell tumors (NSGCT). Pure seminoma is sensitive to radiotherapy and the prognosis is good [6]. However, the prognosis for NSGCT is poor [7], 5-year overall survival rate of mediastinal NSGCT is much lower than that of gonadal NSGCT [4,8]. For decades, complete resection remains stable for patients with GCT. But to the patients with PMMGCT, surgery only has resulted in a poor prognosis. During the last three decades, the clinical outcome of NSGCT has been dramatically improved since the introduction of cisplatin-based chemotherapy. More and more researchers indicate that advanced chemotherapy regimens based on cisplatin have markedly improved the conditions of NSGCT patients [4,8,9]. Nonetheless, many of such patients still have persistent cancer cells in the residual mass after standard cisplatin-based chemotherapy and conventional selvage chemotherapy and seldom results in a long-term disease-free status. Therefore, surgical resection of the residual mass after chemotherapy plays an important role in controlling mediastinal NSGCT. This multimdimensional treatment strategy serves to assess response, to remove chemotherapy-resistant disease, and to guide additional chemotherapy. Controversy still exists regarding the effectiveness between surgical resection and chemotherapy, but multidimensional treatment has become the standard treatment for mediastinal NSGCT. We retrospectively reviewed our 19-year experience with 54 PMMGCT patients to evaluate the efficacy and safety of the multidimensional therapy.

## Patients and methods

### Patients

A medical records database in our department was searched retrospectively to identify patients with PMMGCT. Records for operations and pathology reports were specially reviewed. Between 1990 and 2009, a total of 2152 patients with mediastinal tumors were evaluated at the Department of Thoracic Surgery, Provincial Hospital Affiliated to Shandong University, China. Among these patients, there were 54 patients with continuous PMMGCT, including 47 males and 7 females ranging from 14 to 58 years old (average age was 27 and 28.6 years old respectively). Patients were further divided into seminoma group and NSGCT group. Seminoma mixed with other histologic type was classified as NSGCT.

Fifty-one patients had symptoms at the time of diagnosis: chest pain in 24 cases, dyspnea in 18 cases, cough in 16 cases, fever in 7 cases, hemoptysis in 6 cases, weight loss in 6 cases, back or shoulder pain in 3 cases, superior vena cava obstruction syndrome in 3 cases, and hoarseness in 1 case (Table 1). All male patients had normal testicles.

**Table 1:** Initial symptoms and clinical data in 18 patients with seminoma and 36 patients with nonseminomatous germ cell tumor

	**Seminoma**	**NSGCT**
**Symptom**	**No. of patients (%)**	**No. of patients (%)**
Chest pain	6 (33.3)	18 (50.0)
Dyspnea	3 (16.7)	15 (41.7)
Cough	3 (16.7)	13 (36.1)
Back or shoulder pain	1 (5.6)	2 (5.6)
Hemoptysis	0 (0)	6 (16.7)
Hoarsness	0 (0)	1 (2.8)
Super vena cava syndrom	1 (5.6)	2 (5.6)
Fever	1 (5.6)	6 (16.7)
Weight loss	2 (11.1)	4 (11.1)
Asymptomatic	2 (11.1)	1 (2.8)

NSGCT: nonseminomatous germ cell tumor.

All patients received standard chest X-rays and computed tomography (CT) scans. It showed that all the tumors were located in the anterior or anterosuperior mediastinum. Preoperative histologic diagnosis was carried out in 19 patients by fine needle biopsy guided by ultrasound or CT. As a result, histologic evaluations revealed 7 cases of mediastinal NSGCT, 12 cases of poorly differentiated carcinoma or no diagnosable cells were found. Serum levels of tumor markers, beta-human chorionic gonadotropin (β-HCG) and alpha-fetoprotein (AFP), were tested in 30 patients, among which 7 patients with elevated β-HCG, 15 patients with elevated AFP levels, 4 patients with elevated both β-HCG and AFP levels. Elevated serum in tumor markers (STM) strongly indicated NSGCT. Preoperative elevated levels of β-HCG or AFP and needle biopsy supported the diagnosis of mediastinal NSGCT. The initial clinical diagnosis of mediastinal NSGCT was shown among 31 patients, and other patients were diagnosed as thymoma, thymic carcinoma, and mediastinal tumor, respectively.

## Methods

All statistical analyses were performed with SPSS 10.0 statistical software (SPSS, Chicago, IL, USA). Survival rate was calculated with the Kaplan–Meier method and difference in survival between groups was calculated by using the log-rank test. Differences were considered to be statistically significant when the *P* value was less than 0.05.

### Follow-up

After discharge from the hospital, all patients in this series were evaluated every 3-month for the first 5 years and 6-month intervals thereafter, as well as their clinical history, physical examination, laboratory analysis, CT scan. There were 6 patients out of follow-up. The date of death or last follow-up was defined as the endpoint.

## Results

Among these 54 patients, 52 patients underwent surgical resections, while the other 2 patients only received chemoradiotherapy. Among 52 patients treated with surgery, 22 patients received cisplatin-based chemotherapy as basic treatment on the basis of a serologic diagnosis or the needle biopsy followed by surgical resection; 6 patients received preoperative radiotherapy, which was not very effective, then followed surgical resection thereafter; 24 patients underwent surgery as basic treatment (Table 2). Of all these 52 patients with surgery, 30 patients were treated with complete resection, 18 patients with partial resection and 4 patients had only biopsy. After operation, 42 patients received cisplatin-based chemotherapy and followed by radiotherapy in 14 cases, 5 patients received radiotherapy only. The patients with seminomas were treated with radiotherapy doses ranging from 40 Gy to 50 Gy, while the patients with NSGCT were treated with radiotherapy doses ranging from 50 Gy to 54 Gy.

**Table 2:** Initial therapy of 18 patients with seminoma and 36 Patients with nonseminomatous germ cell tumor

	**Seminoma**			**NSGCT**		
	**No. of patients**	**Complete response or resection**	**Partial response or resection**	**No. of patients**	**Complete response or resection**	**Partial response or resection**
Surgery	8	7	1	16	9	7
Chemotherapy	4	2	2	18	5	13
Radiotherapy	6	2	4	2	0	2

NSGCT: nonseminomatous germ cell tumor.

Surgical incisions have been grouped by the anatomic region and extent of the tumor: median sternotomy in 26 cases, anterolateral thoracotomy in 15 cases and posterolateral thoracotomy in 11 cases. Pulmonary lobectomy was performed in 4 cases and pulmonary wedge resection in 7 cases, partial pericardial resection in 6 cases, ipsilateral phrenic nerve resection in 3 cases, local chest wall resection in 3 cases, superior vena cava reconstruction in 1 case, orthotopic great venous reconstruction with vascular prosthesis placed from the remaining left innominate vein to the superior vena cava-right atria junction in 1 case. These two patients who received the artificial vascular replacement have been surviving free of disease.

There were no perioperative deaths. Major postoperative complications occurred in 9 patients: pulmonary infection in 5 patients, phrenic nerve paralysis in 3 patients, concurrent thrombosis of left innominate vein in 1 patient and was successfully cured by anticoagulant therapy.

The 2 patients who didn’t receive surgery were diagnosed as seminomas via fine needle biopsy. Six histopathologic categories of tumor were found in 52 patients who underwent surgical resection, including 16 pure seminomas, 14 immature teratomas, 11 yolk sac tumors, 3 embryonal cell carcinoma, 3 teratocarcinomas and 5 mixed tumors (yolk sac tumor and immature teratoma in 2 cases, embryonal cell carcinoma and yolk sac tumor in 1 case, yolk sac tumor and seminomas in 1 case and embryonal cell carcinoma and seminoma in 1 case, Table 3).

**Table 3:** Histologic classification of 54 patients with primary mediastinal germ cell tumors

**Tumor type**	**No. of patients**	**Percent (%)**
Seminoma	18	33.3
York sac tumor	11	20.4
Embryonal carcinoma	3	5.55
Immature teratoma	14	25.9
Teratocarcinoma	3	5.55
Mixed germ cell tumor	5	9.3

Follow-up data after surgery were available for 48 patients and 6 patients were out of follow up. At the last follow-up, 17 patients were still alive, including 11 patients with seminoma and 6 patients with NSGCT. Thirty-one patients died within the follow-up period, including 27 patients with tumor-related causes and 4 patients without tumor-related causes.

Of the 16 patients with seminomas treated with surgery, 10 patients were alive (including 8 patients with disease-free survival for over 5 years, 1 patient with a disease-free survival for 46 months and 1 patient with a disease-free survival for 20 months), 5 patients died of tumor-related or other causes and 1 patient was out of follow-up. 2 patients with seminomas were treated only by chemoradiotherapy, 1 patient with a disease-free survival for over 52 months and the other patient with a disease-free survival for over 2 years, but out of follow-up thereafter. Of the 36 patients with NSGCT, 26 patients died of tumor-related causes, 6 patients were alive (including 4 patients with disease-free survival for over 5 years, 1 patient with a disease-free survival for over 28 months, 1 patient was alive for 4 years but with developed disease recurrence) and 4 patients were out of follow-up. Nevertheless, all patients with NSGCT treated with incomplete resection died or lost to follow-up within 2 years. The median survival in the incomplete resection group was 7 months postoperatively (ranging from 4 to 22 months).

**Figure 1:**
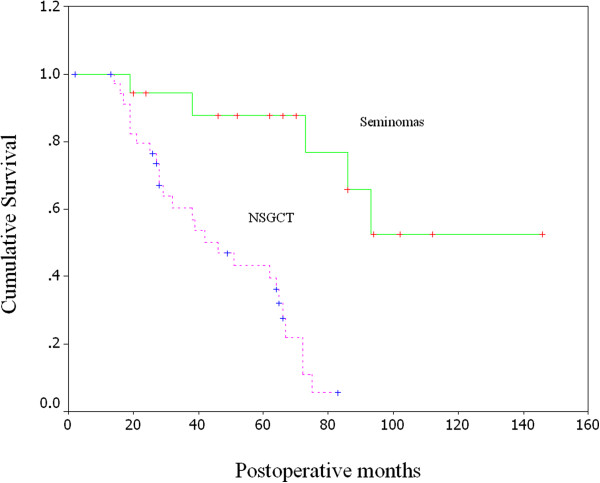
Kaplan-Meier curves of overall survival for 18 patients with seminoma and 36 patients with NSGCT.

The overall 5-year survival rate of the patients with mediastinal seminoma was 87.7%. The overall 3 and 5-year survival rates of the patients with NSGCT were 47.4% and 23.0%, respectively. The prognosis of the patients with seminoma was significantly better than that of the patients with NSGCT (*P* < 0.0001, log-rank test, Figure 1).

## Discussion

The majority of GCT originates from gonad, whereas a minority may occur in the extragonadal places such as mediastinum, retroperitoneum and pineal gland [10]. Primary GCT of the mediastinum is relatively rare and accounts for 10-15% of the mediastinal tumors. While PMMGCT is rather rare and only accounts for 1-4% of the mediastinal tumors [5]. The exact pathogenesis of GCT in the mediastinum is still uncertain. Retroperitoneal germ cell tumors are generally considered to be metastases from primary gonadal lesions, whereas the origin of primary mediastinal and pineal lesions has been a matter of speculation. It is hypothesized that extragonadal germ cell tumor (EGCT) is either a consequence of an abnormal migration of germ cell along the midline from the yolk sac to the embryonic gonadal ridge during embryogenesis or result from germ cells that are distributed physiologically in the liver, bone marrow and brain in order to provide regular functions or convey hematologic or immunologic information [11]. Thus, the midline structures, the anterior mediastinum especially, are the most common occurrence site of EGCT. EGCT can be developed as primary neoplasm in the pineal gland, retroperitoneum, sacral area and hence they may also be in gonadal originallyv [12].

PMMGCT are rare and occur predominantly in young adults [5]. In our cases, male patients account for the vast majority, the mean age at the time of diagnosis was 27 years. PMMGCT are divided into two broad groups, seminomas and NSGCT. Seminomas retain the morphology of spermatogonial germ cells and are extremely sensitive to treatment by radiation as well as chemotherapy [10,13]. NSGCT includes yolk sac tumor, embryonal carcinoma, choriocarcinoma, immature teratoma, terato- carcinoma and mixed tumors with both differentiated and undifferentiated elements [13]. They are, as a group, still sensitive to chemotherapy, although they are less sensitive to radiationtherapy than seminomas [10]. Seminomas are the most common mediastinal malignant GCT [9]. Cesar A et al. reported that primary mediastinal seminomas accounted for approximately 37% of all mediastinal GCT [14] and Clamon and Economon et al. reported that one-half of malignant GCT are seminomas [15,16]. In our series, seminomas occupy 33.3% of all malignant tumors. A higher incidence of NSGCT was found, this may attribute to the cases of immature teratoma. Cesar A reported that yolk sac tumor is the most common NSGCT of the mediastinum, accounting for 12% of all GCT in this location [17]. We retrospectively reviewed 54 cases of continuous PMMGCT in our hospital, including 11 yolk sac tumors.

PMMGCT is often prone to be misdiagnosed, because of their nonspecific clinical symptoms. The presenting symptoms are cough, chest pain, hemoptysis, and/or dyspnea which are second to compression of adjacent tissues. In addition, some patients presented without any symptoms, and the diagnosis was made by a routine physical or radiographic examinations. Sometimes, the diagnosis of PMMGCT may be confused with thymoma, thymic carcinoma and Hodgkin’ disease. In this case, a careful clinical history and the serum levels of β-HCG and AFP may be helpful in making accurate diagnosis. The elevated serum markers of β-HCG and/or AFP will ultimately favor a diagnosis of NSGCT. Takeda S et al. reported that approximately 90% of their patients with NSGCT had elevated STM levels [5]. In our series, the patients with elevated β-HCG and/or AFP are all histologic NSGCT. Some patients were treated with chemotherapy according to the elevated levels of STM. It has also been reported that the STM levels can be survival predictive [18]. Furthermore, the definite diagnosis of PMMGCT relies on pathological examinations. Sometimes, PMMGCT are morphologically indistinguishable from some other malignant tumors, but immunohistochemical studies will ultimately lead to the correct diagnosis. For decades, the preoperative cytologic examination by percutaneous fine-needle biopsy has become the common method to diagnose the mediastinal mass. But sometimes, the needle biopsy samples are too small to do immunohistochemical studies, so the preoperative needle biopsy is usually unreliable. Furthermore, it also increases the possibility of needle track implantation. In our series, 19 patients had needle biopsy, only 7 patients with accurate diagnoses. In this regard, biopsy via mediastinoscopy or thoracoscopy is required for the final diagnosis of highly suspected PMMGCT,but the risk also increases. In addition, GCT could have a mixed histology, so the diagnosis of seminoma based on small biopsy specimens should be considered clinically as well as histopathologically.

Associated syndromes, such as hematologic malignancies (leukemia or myelodysplastic syndrome) have already been reported in some patients with PMMGCT [19]. In addition, NSGCT is occasionally associated with klinfelter syndrome [20]. However, the reasons for these associations in mediastinal NSGCT remain to be clarified. We didn’t notice such associated syndromes in any of our cases.

In the last two decades, several authors have reported the effectiveness of cisplatin-based chemotherapy, which has become the standard therapy for PMMGCT [4,8,9]. The role of surgical resection has been changed into multidimensional therapy for PMMGCT. Recently, the treatment of PMMGCT with cisplatin-based chemotherapy followed by surgical resection of residual disease is currently one of the most successful approaches of multidimensional therapy [18].

We currently believe that most of the patients with PMMGCT can not be diagnosed definitely before operation, but only can be assured by intraoperative biopsy or resected specimens. Primary surgical resection is still the most important way. Particularly to the patients with small and resectable tumors without any invasion, we should first perform thoractomy for radical resection, followed by postoperative chemotherapy and/or radiotherapy. In fact, patients really benefit from surgery when complete excision can be performed, but partial resection has not been demonstrated beneficial [21]. In our cases, partial resection or partial response to chemotherapy resulted in a subsequent rapid progression of the disease. Therefore, complete resection must be the surgical target in every case. But to the patients with locally advanced tumors, which involved adjacent organs and couldn’t be completely resected, systematic chemotherapy could be recommended to reduce the tumor mass and prevent metastases outside the mediastinum, if necessery, selvage resection could be performed thereafter.

It is well known that seminomas are radiosensitive tumors [6]. Primary or adjuvant radiations for mediastinal seminomas have been effective in the local control of the tumors [4,9,15]. Based on our observations, it appears that complete surgical resection of mediastinal seminomas followed by local radiation and cisplatin-based combination chemotherapy would be the very effective treatment for these patients. Nevertheless, NSGCT are relatively resistant to radiotherapy which is not recommended. To the patients of NSGCT with large mediastinal mass, aggressive cisplatin-based chemotherapy followed by resection of the residual tumor has become the best approach to improve the survival of these patients. This approach avoids demanding or incomplete resections and unnecessary resections. Thoracic surgery was typically delayed for 4 weeks after chemotherapy, which allowed the patient, in particular, to have the bone marrow to recover.

Most of the patients with PMMGCT have large mediastinal mass, even extensive involvement of the adjacent organs. Not infrequently, the mediastinal mass is adherent to the mediastinal surfaces of the cardiac chambers, surgery is technically demanding. Nonetheless, we should perform aggressive resection in the patients with PMMGCT if anatomically feasible, including, if necessary, the artificial vascular replacement,great vein and alternative cardiac chamber resection. In our cases, the two patients who had been performed the artificial vascular replacements had a better prognosis. Although the success of chemotherapeutic regimens in PMMGCT is important, skilled thoracic surgery is an equally important component for successful multidimensional therapy. In this series, patients with PMMGCT who underwent initial tumor resection had higher complete response rate and disease-free survival compared with those who had only biopsy or partial resection. But the role of initial tumor resection needs further evaluation in prospective studies.

Wright CD et al. had emphasized the importance of normalization of STM before surgical extirpation of residual disease in patients with NSGCT [22]. The patients whose STM remained elevated after first-line chemotherapy should receive second-line chemotherapy. But Kesler KA et al. [20] reported that operable patients should undergo surgical extirpation of residual disease after first-line chemotherapy, regardless of STM status. In our series, the STM levels were not measured in some patients before or after surgery,more experiences would be required to evaluate the role of surgery in patients with an elevated and increasing STM before surgical resection.

In conclusion, the prognosis of patients with seminomas is significantly better than that of patients with NSGCT. The results of multidimensional therapy for PMMGCT depend on both successful chemotherapy and surgery. The complete resection of the tumor is important, with wide surgical margins including great vein and adjacent structures, if necessary. New therapeutic strategies are currently being studied in the treatment of the patients with PMMGCT to minimize operative morbidity and to improve the long-term survival equivalent to that of testicular NSGCT.

## Competing interests

The authors declare that they have no competing interests.

## Authors’ contributions

YL, ZW and YY conceived the study idea and designed the study. YL, ZW and YY reviewed the literature and performed statistical analyses. YL, ZW, ZMP and YY drafted the manuscript. YL and YY reviewed and edited the manuscript. All authors read and approved the final manuscript.
